# Mapping Phenotypic Information in Heterogeneous Textual Sources to a Domain-Specific Terminological Resource

**DOI:** 10.1371/journal.pone.0162287

**Published:** 2016-09-19

**Authors:** Noha Alnazzawi, Paul Thompson, Sophia Ananiadou

**Affiliations:** National Centre for Text Mining, Manchester Institute of Biotechnology, Manchester University, Manchester, United Kingdom; Hopitaux Universitaires de Geneve, SWITZERLAND

## Abstract

Biomedical literature articles and narrative content from Electronic Health Records (EHRs) both constitute rich sources of disease-phenotype information. Phenotype concepts may be mentioned in text in multiple ways, using phrases with a variety of structures. This variability stems partly from the different backgrounds of the authors, but also from the different writing styles typically used in each text type. Since EHR narrative reports and literature articles contain different but complementary types of valuable information, combining details from each text type can help to uncover new disease-phenotype associations. However, the alternative ways in which the same concept may be mentioned in each source constitutes a barrier to the automatic integration of information. Accordingly, identification of the unique concepts represented by phrases in text can help to bridge the gap between text types. We describe our development of a novel method, *PhenoNorm*, which integrates a number of different similarity measures to allow automatic linking of phenotype concept mentions to known concepts in the UMLS Metathesaurus, a biomedical terminological resource. PhenoNorm was developed using the PhenoCHF corpus—a collection of literature articles and narratives in EHRs, annotated for phenotypic information relating to congestive heart failure (CHF). We evaluate the performance of PhenoNorm in linking CHF-related phenotype mentions to Metathesaurus concepts, using a newly enriched version of PhenoCHF, in which each phenotype mention has an expert-verified link to a concept in the UMLS Metathesaurus. We show that PhenoNorm outperforms a number of alternative methods applied to the same task. Furthermore, we demonstrate PhenoNorm’s wider utility, by evaluating its ability to link mentions of various other types of medically-related information, occurring in texts covering wider subject areas, to concepts in different terminological resources. We show that PhenoNorm can maintain performance levels, and that its accuracy compares favourably to other methods applied to these tasks.

## Introduction

Human phenotypic information constitutes the observable traits of human beings (e.g., height, eye colour, etc.) resulting from genetic make-up and environmental influences. A more contemporary definition of phenotypes includes the measurable biological, behavioural or cognitive markers that distinguish individuals with specific medical conditions from the general population [1]. In the context of this article, the term *phenotypic information* refers specifically to the causes, risk factors, signs or symptoms of a given disease.

Detailed information about phenotype concepts relating to different diseases can be found in documents from various sources with different focus and perspective, e.g., narrative reports within EHRs and scientific literature articles. Narrative EHR information includes details about individual patient diagnoses, medication, family history, patient past history, signs, symptoms and findings, whilst scientific articles tend to summarise the latest research findings, results and advances in knowledge relevant to different diseases [2, 3]. Given that these different types of information can often be complementary to each other, important details may be overlooked if only a single source (or *text type*) is considered. As such, automated methods to combine relevant details from different text types can be extremely useful, not only to discover extended information about a given concept (e.g., to gather alternative perspectives regarding risk factors contributing to a given disease), but also to uncover novel associations between diseases and phenotypes, which may be scattered amongst documents, both within a given text type and across different text types.

An important means of establishing links between information contained within different documents is to determine when certain types of information are shared, e.g., those documents that mention a common concept. However, this can be problematic, according to the many possible ways in which a given concept can be mentioned in text. For example, *rheumatoid arthritis*, *RA* and *atrophic arthritis* are examples of ways in which the same disease concept could appear in text. The types of variability amongst concept mentions may also be dependent on the characteristics of the text type. For example, scientific literature articles constitute formal text conforming to conventions of structure and readability, whereas the narrative content within EHRs, intended to be used only in a hospital context by doctors, often exhibits a proliferation of undefined (and partly ad hoc) short forms, e.g., acronyms and abbreviations, and there are typically many spelling and/or grammar mistakes [3].

The automatic identification and classification of words and phrases describing important concepts is carried out by a process known as *Named Entity Recognition (NER)*. Each of these words and phrases (subsequently referred to as *entity mentions*) is assigned a label or *semantic category* from a pre-defined set, to characterise the type of concept being described, e.g., *gene*, *disease*, *symptom*, etc. NER systems have been customised for both literature and narrative clinical text [4–9], and their accuracy has improved greatly over the last decade.

To allow different types of information about a given concept to be gathered/combined, possibly from multiple documents belonging to heterogeneous text types (e.g., narratives in EHRs and literature articles), it is important to associate each entity mention in a document with the unique concept that it represents. Links can then be established between entity mentions that describe the same concept in different ways, both within and across documents belonging to different text types.

Typically, each entity mention is automatically linked or *mapped* to a concept entry in a terminology, ontology or thesaurus (henceforth referred to collectively as *terminological resources*), which provides a comprehensive inventory of information about domain-specific concepts. According to recent conventions, we refer to this process as entity *normalisation*. Terminological resources usually assign a unique identifier to each concept, and provide various other types of information about the concept, such as a textual or formal definition, an account of semantic relationships with other concepts, and a listing of several terminological units, i.e., single or multi-word phrases that are frequently used to refer to the concept in text, including acronyms and abbreviations [10]. We subsequently refer to these terminological units as *(concept) synonyms*.

Normalisation methods usually work by trying to match each entity mention in a document with a concept synonym in a terminological resource. This allows the entity mention to be associated with the concept under which the synonym is listed in the terminological resource. A potential issue is that terminological resources are typically manually curated and not primarily designed for use by automatic entity normalisation methods. Thus, they do not attempt to exhaustively account for *all* possible ways of mentioning a concept in text. Indeed, given the often highly creative nature of language, compiling such a list would be virtually impossible. Accordingly, automatic normalisation methods propose different ways to find the most accurate mapping between an entity mention in text and a concept synonym in a terminological resource, when no exact match can be found.

To the best of our knowledge, no previously reported automatic normalisation method has focussed specifically on phenotype concepts. However, this represents a particularly challenging task, due to the diverse ways in which concepts describing phenotypes can be mentioned in text. Such diversity is particularly prevalent within narrative EHR reports. In this article, we report on our development of a novel method (*PhenoNorm*), specifically designed to normalise phenotype entity mentions in heterogeneous text types to concepts in the UMLS Metathesaurus [11], a large and widely-used repository of biomedical terminology. To address the complexity of the task, *PhenoNorm* integrates a number of different string-based and semantic similarity measures to allow flexible, accurate normalisation of phenotype entity mentions with a range of different internal structures/characteristics.

PhenoNorm was developed with the aid of a pre-existing text corpus, PhenoCHF [12], whose documents (both narrative EHR reports and biomedical literature articles on the subject of congestive heart failure (CHF)) are *annotated* (i.e., marked up) by medical experts with phenotypically-focussed entity mentions belonging to a number of semantic categories. As such, PhenoCHF provides valuable evidence about how phenotype concepts can be mentioned in text, and how these mentions vary in different text types.

We have evaluated the performance of PhenoNorm in normalising phenotype mentions in PhenoCHF, and we show that it achieves higher accuracy than other, more general normalisation methods when they are applied to the same task. We have also conducted a number of additional experiments to demonstrate that PhenoNorm is a highly flexible method, which performs robustly and consistently when used to normalise entity mentions belonging to a range of semantic types, in documents belonging to different text types and covering alternative biomedical subject areas. This is particularly the case when normalisation is carried out to the same terminological resource, although good performance can still be achieved when a different terminological resource is used. We furthermore demonstrate that when applied to these alternative normalisation tasks, PhenoNorm is still able to perform competitively with alternative methods.

The remainder of this article is organised as follows. Firstly, in the *Related Work* section, we review a number of areas of research that are highly relevant to our own work. These include previous efforts to recognise and normalise entity mentions in biomedical text, as well as recent work to recognise phenotype-related information in text. Subsequently, in the *Methods* section, we describe the work involved in designing our novel PhenoNorm method, and provide a detailed description of the different steps involved in the final algorithm. In the *Results* section, we firstly report and analyse the normalisation results produced by PhenoNorm, and compare these with a number of baseline methods applied to the same task. We then provide a more detailed analysis of the different types of concepts that are mentioned in narrative EHR reports and literature articles, and finish this section by assessing the performance of PhenoNorm when applied to other normalisation tasks. Finally, in the *Conclusion* section, we summarise our contribution and results, and we provide some directions for future work.

## Related Work

As mentioned above, NER is an important first step to determine which phrases in text correspond to concept mentions. Approaches to NER may be largely divided into *terminology-driven* and *corpus-driven* approaches. In the former, normalisation of entity mentions occurs as an integral part of the NER process, whilst the latter normally require a separate normalisation step, thus motivating the need for dedicated normalisation methods, such as PhenoNorm. Depending upon the subject of the text and the purpose of the normalisation, a large variety of terminological resources could be used to provide the target concepts. For example, comprehensive resources are available that concentrate on specialised types of terminology, e.g., gene-related information [13], diseases [14, 15], clinical care [16, 17] and phenotypes [18, 19], as well as larger-scale resources that cover a wider range of biomedical and health-related concepts (e.g., the UMLS Metathesaurus [11], which integrates a large number of different terminologies, including several of the above, such as those relating to phenotypes).

### NER approaches

In terminology-driven NER systems, the process of finding entity mentions is primarily driven by matching words and phrases in text with concept synonyms in a terminological resource. As such, the normalisation of entity mentions to concepts is an integral part of the NER process, and a separate normalisation step is not required.

The earliest terminology-driven NER methods used strict matching between phrases in the text and concept synonyms listed in the associated resource (e.g., [20]). Subsequent methods introduced simple ways to better account for the variable nature of language, such as removing inflections (e.g., plurals) [21], whilst later approaches (e.g., [22–28]) have employed a greater variety of heuristics to try to account for the potential multitude of ways in which concepts can be mentioned in text. These include ignoring certain words that occur in entity mentions but not in concept synonyms (e.g., articles, pronouns, etc.), generating derivations of words in concept synonyms to match more entity mentions (e.g., *elevate -> elevation*), using additional lexical resources, generating permutations of words in concept synonyms (e.g., *increase in blood pressure -> blood pressure increase*) and disambiguating mentions with several potential concept mappings, according to surrounding text (e.g., the entity mention *MI* could correspond to *myocardial infarction* or *mitral incompetence*). Linguistic pre-processing ensures that only linguistically meaningful units (e.g., noun phrases) are considered as potential concept mentions. Partly as a consequence of the frequent update of certain underlying terminological resources (e.g., the UMLS Metathesaurus) to include new concepts and synonyms, such approaches remain popular. This is evidenced by the continuing evolution and improvement of very mature tools (e.g., MetaMap [29]) and the appearance of new methods that follow the same general approach (e.g., [30]).

The major drawback of terminology-driven approaches is their primary reliance on manually curated (and hence, incomplete) terminological resources. Regardless of the heuristics applied, only entity mentions that match (or closely resemble) synonyms listed in the resource will be recognised and normalised.

Corpus-driven approaches to NER tackle the problem from a different angle, by using evidence from text corpora to derive general linguistic patterns that signify the presence of entity mentions, usually using sophisticated machine learning methods. Corpus-driven methods usually have less (if any) reliance on information in terminological resources, and according to their employment of generalised patterns to recognise entity mentions, they can often recognise a wider range of entity mentions than terminology-driven approaches.

Despite the advantages of corpus-driven methods, there are some potential issues. The first of these is their usual reliance on *annotated corpora*, in which experts must meticulously mark-up mentions of relevant concept types in a collection of documents; such corpora require much time and effort to produce. Secondly, since corpus-driven NER methods are usually largely detached from terminological resources, the entity mentions that these methods recognise may have forms that vary considerably from associated concept synonyms listed in terminological resources. This means that dedicated normalisation methods are usually required to “bridge the gap” between the ways in which concepts are actually mentioned in text and how they are represented in the terminological resource.

Despite the difficulties faced in creating annotated corpora, numerous collections of biomedical literature have been annotated with molecular-level entities (e.g. genes and proteins) and diseases, either by individual research groups (e.g., [31–34]), or more recently in the context of *shared tasks* (e.g., [35–41]), which encourage several research teams to focus their attention on challenging natural language processing (NLP) problems. When developed in the context of shared tasks, the production costs of annotated corpora are offset by the high likelihood that they will act as a driver for advancing research, and that they will be widely used by the NLP community. Although annotated corpora of narrative clinical text are scarcer, largely according to privacy concerns in relation to their potentially sensitive content [7], efforts to stimulate rapid advances in clinically-focussed NLP methods through shared tasks [42] have resulted in the organisation of a number of shared task series, e.g., i2b2 [5, 7, 43, 44] and the ShARe/CLEF eHealth Evaluation Labs [45–47]. The ShARe/CLEF corpus of narrative clinical reports released for Task 1 of the 2013 Lab [45], annotated with mentions of disorders, each mapped to an appropriate concept in the UMLS Metathesaurus (henceforth referred to as *the ShARe/CLEF corpus*), has also formed the basis for further shared tasks [48, 49].

### Normalisation of entity mentions

The need for sophisticated normalisation methods to handle entity mentions recognised by corpus-driven approaches is clearly demonstrated in [50], in which an assessment of the ability of four widely-used, terminology-driven tools to carry out recognition and normalisation of disorder entities, using the ShARe/CLEF corpus as a gold standard, demonstrated poor performance by all of them. However, the increasing emergence of annotated corpora that include gold-standard links between entity mentions and concepts in terminological resources [33, 34, 45] has stimulated a large amount of research into the development of dedicated normalisation methods for both biomedical scientific text and narrative clinical text.

The majority of normalisation methods are based on matching entity mentions against concept synonyms listed in a terminological resource (e.g., [22, 23, 51–53]); more sophisticated methods combine or rank the results obtained using a number of different terminological resources [54, 55]. Approaches based on pattern-matching or regular expressions (e.g., [56–58]) can account for frequently occurring variations not listed in the terminological resource (e.g., Greek or Roman suffixes for genes) and/or by helping to post-process initial normalisation output [59], in order to better handle problematic cases such as abbreviations or coordinated phrases [60]. Methods based on machine learning, or hybrid methods combining rules and machine learning, have also been proposed (e.g., [61, 62]).

*String similarity methods* have also been employed in a number of normalisation efforts (e.g., [63, 64]). They assign a numerical score representing the degree of similarity between an entity mention and a concept synonym, which means that, unlike the limited types of variations that can be handled by rules or regular expressions, string similarity methods can handle a virtually unlimited range of variations. Character-level methods consider the number of edits (e.g., insertions, deletions or substitutions) required to transform one phrase into another (e.g., [65]), or look at the proportion and/or ordering of characters that are shared between the phrases being compared [65–67]. This can help to account for the fact that concepts may be mentioned in text using words that have the same basic root but many different forms, including different inflections (e.g., *reduced* vs. *reduce*), alternative spellings (e.g. *oedema* vs. *edema*) and nominal vs. verbal forms (e.g., *reduce* vs. *reduction*).

Word-level similarity metrics (e.g., [68]) can be more appropriate when the phrases to be compared consist of multiple words. Such metrics make it possible to ensure that a match is only considered if a certain proportion of words is shared. Weights may be applied to the individual words (as is the case for TF-IDF (Term Frequency-Inverse Document Frequency)[69]), to ensure that greater importance is placed on matching words with high relevance to the domain, than function words like *the*, *of*, etc.

Hybrid methods (e.g. SoftTFIDF [70]) also operate at the word level, but use a character-based similarity method to allow matches between words that closely *resemble* each other, even if they do not match exactly. This helps to account for the fact that concepts may be mentioned in text using multi-word terms whose exact forms may vary from synonyms listed in the terminological resource. Such methods can also help to address the problem of normalising entity mentions containing spelling errors, which are very frequent in clinical narrative text (e.g., to allow mapping of the entity mention *high blood presser* to the concept synonym *high blood pressure* [69]).

Despite their flexibility, string similarity methods can only handle *surface-level* variations. If an entity mention has a similar *meaning* to concept synonym, but it uses words whose appearance is different to those used in the concept synonym, then is likely that an incorrect mapping will be assigned. An example is the entity mention *worsening in exercise tolerance*, whose closest concept synonym in the UMLS Metathesaurus is *reduced exercise tolerance*. String similarity methods will typically map entity mentions only to those concept synonyms in which all words match or look similar to words in the entity mention. In the case that the UMLS Metathesaurus is used as the terminological resource, this would result in a mapping to the more general concept synonym *exercise tolerance*. Accordingly, the accuracy of string similarity methods could be improved by integrating semantic-level information.

### Acquiring phenotype knowledge from text

There has been increasing interest in the automatic acquisition of knowledge about phenotypes from text. This has been partly driven by the *Phenotype day* events [71, 72], which have the overarching aim of bringing together researchers with different backgrounds to support the process of deep phenotyping. Topics covered have included creating ontologies to represent phenotypes [73–75], developing tools and pipelines to support phenotypic data curation and integration with ontologies [55, 75, 76], and applying phenotype knowledge acquisition in real world applications (e.g., discovery of phenotype-genotype relations) [77–79].

Efforts to create corpora that are focussed specifically on annotating mentions of phenotype-related concepts have included a silver-standard (i.e., automatically annotated) corpus [50], which combined the outputs of several tools to ensure higher quality annotations, as well as a number of gold standard corpora concerning the annotation of mentions of phenotype concepts concerned with specific diseases (i.e., COPD [55], CHF [12], heart failure, rheumatoid arthritis and pulmonary embolism [73]). Of these, only the latter 3 corpora (all developed as part of the same research effort) include normalised annotations, i.e., entity mentions are linked to concepts in the UMLS Metathesaurus. In all of these phenotype-centric annotated corpora, fine-grained semantic categories such as *Cause*, *Risk Factor*, *Individual Behaviour* and *Sign or Symptom* provide a more detailed encoding of phenotype information. However, the fact that several of these categories do not correspond directly to semantic categories in the UMLS Metathesaurus motivates need to develop customised methods to recognise and normalise phenotype entity mentions. Whilst in our previous work, we reported on our efforts to develop NER methods to recognise phenotype entity mentions relating to CHF, we are not aware of any previous research that has specifically targeted the normalisation of phenotype entity mentions.

## Methods

In this section, we describe how the design of our PhenoNorm method began with a preparatory analysis of entity mentions previously annotated in a phenotype-focussed corpus, i.e., PhenoCHF, to try to determine the range of ways in which phenotype concepts can be expressed in text. As a result of this initial analysis, we set down a number of important considerations for the design of a normalisation method for phenotype entity mentions. Finally, we provide a detailed account of the steps involved in the final PhenoNorm method, and we describe how the design considerations have been fulfilled within the method.

### Analysis of phenotype mentions in the PhenoCHF corpus

The PhenoCHF corpus [12] provides evidence of how a common set of phenotype concept types is mentioned in heterogeneous text types (i.e., narrative EHR reports and biomedical literature). The EHR part consists of 300 discharge summaries (a subset of the documents from the i2b2 recognising obesity challenge [5]), while the literature part consists of 10 full text literature articles. In both cases, documents are concerned with patients suffering from CHF as a major complaint, as well as from kidney failure. It has previously been shown that the corpus can be used successfully to train NER methods that can robustly recognise phenotype entities in heterogeneous text types [4].

All documents in PhenoCHF were manually annotated by medical experts for phenotypic information related to CHF. The annotation includes entity mentions relating to four semantic categories of phenotype-related concepts, as shown in Table 1.

**Table 1:** Types and statistics of entity mentions annotated in the PhenoCHF corpus.

Semantic categories	Description	# of annotated mentions in narrative EHR reports	# of annotated mentions in literature articles
**Cause**	Any medical problem that contributes to the occurrence of CHF	1320	1107
**Risk factor**	A condition that increases the chance of a patient having CHF	1335	408
**Sign or symptom**	Any observable manifestation of a disease. Symptoms are subjective manifestations experienced by a patient and reported to a health professional. Signs are physical manifestations of a disease observed by someone other than the patient, e.g. a physician using by physical examination of diagnostic tests.	2449	304
**Non-traditional risk factor**	Conditions associated with abnormalities in kidney functions that put a patient at higher risk of developing *signs or symptoms* and *causes* of CHF	308	329

An examination of the entity mentions annotated in PhenoCHF reveals that phenotype concepts can be mentioned in text using varying syntactic structures of different lengths, including simple noun phrases (e.g. *progressive renal failure*), coordinated noun phrases (e.g., *increased chest pain and fatigue*), noun phrases followed by prepositional phrases (e.g., *increasing dyspnea on exertion*) and complete clauses or sentences (e.g., *jugular venous pressure is elevated*). Phenotype mentions in narrative EHR reports exhibit a balanced distribution between these different syntactic structures, whilst entity mentions in scientific articles usually correspond to noun phrases.

The diversity of entity mentions exhibited in PhenoCHF would prove problematic for a number of terminology-driven NER/normalisation methods, since they are usually only able to handle entity mentions expressed as simple noun phrases. Furthermore, the concept synonyms listed in the UMLS Metathesaurus often have rather different forms to the entity mentions encountered in PhenoCHF. Specifically, synonyms in UMLS tend to constitute noun phrases, which are frequently provided in a standardised form that does not typically reflect how the concept is actually expressed in running text, e.g., the UMLS synonym *Family history*: *myocardial infarction* may appear in text as *father died of a myocardial infarction*.

### Design of a novel phenotype normalisation method

The annotated entity mentions in the original version of PhenoCHF did not include gold standard links to concepts in a terminological resource. Indeed, at the time that we developed PhenoNorm, we were not aware of any phenotype-focussed annotated corpus with gold-standard normalisations of entities. Although the corpora reported in [73] have subsequently been released, which do include gold standard normalisation of entity mentions to UMLS concepts, these corpora only include formal text. However, as our analysis of PhenoCHF has shown, the highly variable ways in which concepts can be mentioned in narrative clinical text means that it is important to account for these in developing an effective and robust normalisation method for phenotype entity mentions.

Without gold standard normalisation annotations, however, we could not develop a method based on machine learning techniques. Instead, we chose to develop a hybrid method, which integrates various similarity methods to specifically address the range of variability in entity mentions that are evidenced in PhenoCHF. We determined that our novel method should take the following into account:

- **Variability**—differences between entity mentions in text and concept synonyms in the UMLS Metathesaurus are particularly prevalent for phenotype entity mentions, e.g., variations in word order and internal structure of entity mentions, different word forms, etc. The flexibility offered by string similarity methods makes them far more suitable than rules in this context.
- **Specificity**—entity mentions should be mapped to concepts with the correct level of specificity. A large number of UMLS concepts correspond to qualified versions of more general concepts. Therefore, if an entity mention includes a *qualifier*, it should normally be mapped to a *qualified* UMLS concept. For example, the mention *elevated* *jugular venous pressure* should be mapped to the concept synonym *raised* *jugular venous pressure*. Even though the qualifier terms used in the mention and the concept synonym are different, it is important that, in contrast to existing methods, our novel method should not simply map the entity mention to the shortest concept synonym with the greatest number of shared words (in this case *jugular venous pressure*), since this represents a more general concept.
- **Semantic-level variations**—Entity mentions may include not only different *forms* of words compared to those contained within UMLS concept synonyms, but also words with closely related meanings. For example, the entity mention *elevated pulmonary capillary wedge pressure* should be mapped to the concept synonym *pulmonary capillary wedge pressure increased*. However, using a string similarity (edit distance) approach, an incorrect concept synonym (*pulmonary capillary wedge pressure decreased*) will be chosen.

### PhenoNorm method

Our novel PhenoNorm method takes into account all of the above design considerations, as detailed below in our description of the steps that are carried out by the main algorithm. However, we firstly describe a number of pre-processing steps that were carried out in an attempt to improve the accuracy of the mappings:

- We created a filtered version of the UMLS Metathesaurus, to ensure that our normalisation process would not consider irrelevant UMLS concepts (i.e., those not corresponding to phenotypic information). The Metathesaurus organises its large number of individual semantic categories into a smaller number of higher-level semantic groups. Although the phenotype entity categories used in PhenoCHF do not correspond to individual UMLS semantic categories, they can all be considered to fall within the scope of the more general *disorder* semantic group (which includes categories such as *Disease or Syndrome*, *Mental or Behavioral Dysfunction* and *Finding*). This has been confirmed both by the PhenoCHF annotators and in previous studies (e.g., [80]). We thus filtered the UMLS vocabulary to retain only those terms that belong to this semantic group. We then built an inverted index, which, for each annotated entity mention, links each word in the mention to all UMLS concept synonyms in which it occurs.
- We observed that a large number of annotated entity mentions in PhenoCHF take the form of co-ordinated noun phrases, which actually correspond to mentions of two or more distinct UMLS concepts, e.g., the annotation *increased chest pain and fatigue* mentions two different UMLS concepts, i.e., *increased chest pain* and *increased fatigue*. To address this, we applied an existing rule-based module (Baumgartner et al. [81]), which uses information from a linguistic analyser [82] to split coordinated phrases appropriately into separate entity mentions.
- Stop words (e.g., *the*, *is*, *was*, etc.) were removed from annotated entity mentions, and abbreviations were expanded into their full forms with the aid of MetaMap.

**Fig 1:**
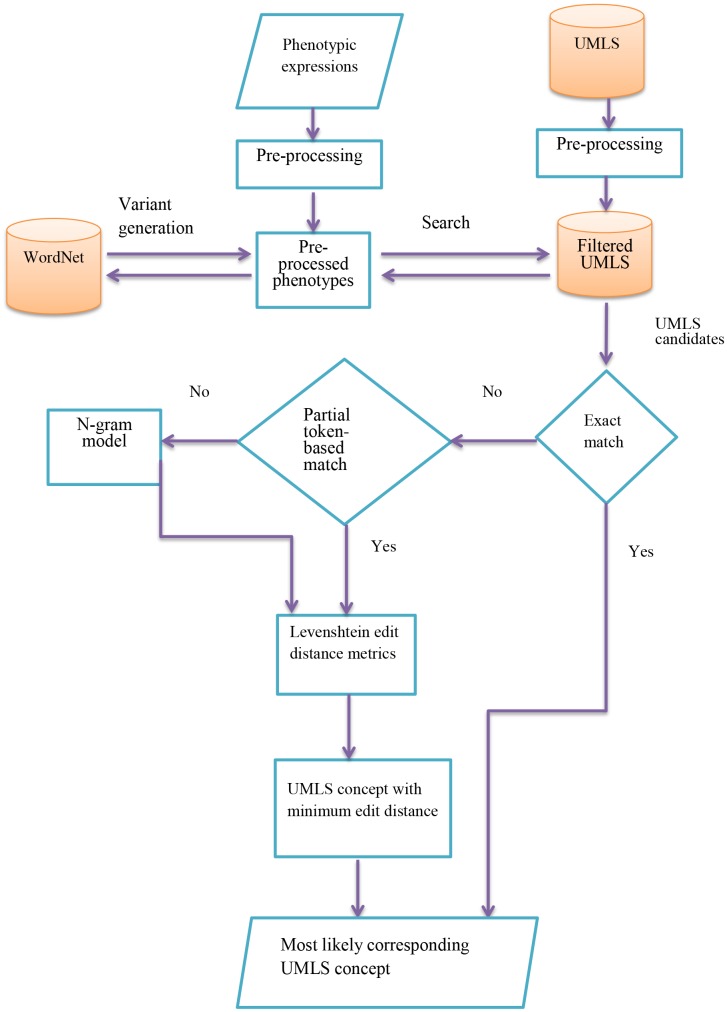
PhenoNorm normalisation workflow.

Following the pre-processing stages, the following steps were undertaken for each phenotype entity mention, as summarised in Fig 1.

1. Semantic-level variants of the mention were generated, with the aid of the WordNet lexical database of English [83]. In this large resource, words are organised into sets of synonyms, called *synsets*, which are linked together into a semantic network, using different types of relations. For example, the words *elevated* and *raised* occur in the same synset, which is linked to the synset that contains the word *increased*, via the *similar to* relation. Potential semantic variants of each entity mention were obtained by using WordNet to find synonyms and closely related words for each adjective or noun appearing in the original mention. All possible combinations of semantic variants were then generated, resulting in a set of phrases (including the original entity mention), each consisting of *n* tokens (*token(1)*,*token(2)……token(n)*).
2. For each *token(i)* in each mention/variant, the inverted index is consulted, and all UMLS concept synonyms in the filtered list that contain the token are retrieved.
3. Candidate UMLS concept synonyms with the most similar sets of tokens to the phenotype mention/variant are found by computing the intersection between the sets of hits retrieved for each *token (i)*. This set of candidates is then reduced by considering only those whose tokens match most closely to those in the mention/variant. If any of the candidate UMLS synonyms matches exactly with the phenotype mention/variant, then the algorithm terminates. Otherwise, the closest non-exact matches are sought. Firstly, it is determined whether any of the candidate UMLS synonyms shares all words with the mention/variant (but possibly in a different order). If such candidates exist, then the algorithm moves on to step 4. If no such candidates exist, then the constraint is relaxed, such that candidates with only *(n-1)* matching words will be considered, and so on. Note that this step will potentially retain UMLS synonyms with words that do not match words in the entity mention. This can help to increase the likelihood of a correct mapping when neither a mention nor its generated semantic variants correspond exactly to a concept synonym. For example, given the mention *heightened blood pressure*, the concept synonym *high blood pressure*, corresponding to the correct qualified concept, will be retained as a potential candidate.
4. Each candidate UMLS synonym identified in step 3 is assigned a score based on its level of similarity to the mention/variant. This score is calculated according to both the Levenshtein distance and the overall difference in the length of the mention/variant and the candidate UMLS synonym. Length is considered to be important, especially when considering qualified concepts, since it would normally be expected that mentions of qualified concepts will have similar overall lengths to concept synonyms listed in the resource. We chose to use the Levenshtein distance metric, according to its flexibility in taking into account different types of character-level operation (insertion, deletion, replacement) that may be required to transform one phrase into another, and also based on its successful application in other normalisation efforts [84, 85].
5. The phenotype mention is mapped to the UMLS concept associated with the synonym that achieves the lowest score from step 4 when compared to either the original phenotype mention or one of its semantic variants. The lowest score indicates the highest degree of similarity between the phenotype mention/variant and UMLS synonym.
6. If the phenotype mention does not contain any tokens that match with a UMLS synonym (e.g.,*diabetesmellitus*, whose closest UMLS synonym is *diabetes mellitus*), then character n-grams (which have been used in previous normalisation efforts e.g., [86]) are employed as the means of retrieving candidate UMLS synonyms (where *n = 5* by default, and *n = 3* if the length of the *token (i)* is less than 5). For each *token (i)* in the phenotype mention, all UMLS synonyms containing the least frequent (rarest) n-gram in *token (i)* are retrieved, since rare n-grams tend to be the most informative. Steps 3–5 are then repeated.

## Results

To allow the performance of the PhenoNorm method to be evaluated, we created an enriched version of the PhenoCHF corpus, with gold standard normalisations, i.e., links between each annotated entity mention and its corresponding UMLS concept, which are verified by a domain expert. We created this mapping in a semi-automatic manner, in order to reduce the time and effort required on the part of the medical expert annotator. In this respect, we follow a similar approach to [73], where it was shown that this process can result in high quality annotation. We firstly applied PhenoNorm to the complete PhenoCHF corpus, and then asked the medical expert to verify or correct the mappings identified. The expert was one of the annotators of the original PhenoCHF corpus, and so was already very familiar with the characteristics of the corpus and the nature of the task.

To verify the utility of PhenoNorm, we compared its performance to that of a range of previously proposed “baseline” normalisation methods, when applied to the same task. We compare both terminology-driven and string similarity methods, which have previously been applied to the task of normalising entity mentions in clinical narrative text [50, 87].

We have applied two terminology-driven baselines, i.e., the mature and highly used MetaMap [22], and the more recently released BeCAS [30], both of which firstly split the input text into sentences, and then identify the noun phrases in each sentence. Entity mentions are found by matching these noun phrases against concept synonyms in the UMLS Metathesaurus. Heuristics are employed to allow non-exact matches between entity mentions and UMLS synonyms, i.e., MetaMap generates potential variants while BeCAS uses regular expressions. Both tools were configured to recognise (and normalise) mentions of concepts falling under the *disorder* semantic group, according to our previous observation that mentions of phenotypes will normally fall under this group.

As the string similarity baseline method, we selected SoftTFIDF [70], according to the common features that it shares with PhenoNorm, i.e., it combines both token-level and character-level similarity measures. We used the implementation of this method provided in the secondstring package [70], which has been shown to perform well across several different string-matching problems (e.g., record linkage) [70].

To evaluate the normalisation performance of PhenoNorm and the other baseline methods when applied to the PhenoCHF corpus, we follow the procedure employed in a closely related task (i.e., the entity normalisation task in the 2013 ShARe/CLEF eHealth Evaluation Lab) in that we report the results in terms of *accuracy*. This is calculated as follows:

Accuracy= correct/total

Where *correct* refers to the number of entity mentions that are normalised to the correct UMLS concept, while *total* refers to the total number of entity mentions in the corpus.

For both PhenoNorm and the baseline methods, we provide in-depth analyses of the normalisation results, including some specific examples of cases where the method in question is able to produce correct normalisations. For each method, we also highlight some problematic cases.

### PhenoNorm performance evaluation

Detailed evaluation results of applying PhenoNorm to the PhenoCHF corpus are summarised in Table 2. To assess the contribution of the WordNet-driven generation of semantic variants of entity mentions, we compare the results obtained both with and without the application of this step.

**Table 2:** Results of applying PhenoNorm to the PhenoCHF corpus.

Phenotypic categories	Accuracy	
	Without post-processing	With post-processing
**EHRs**		
**Cause**	0.899	0.907
**Risk factor**	0.745	0.759
**Sign or symptom**	0.789	0.835
**Non-traditional risk factor**	0.869	0.887
**Average**	0.825	0.847
**Articles**		
**Cause**	0.917	0.917
**Risk factor**	0.878	0.889
**Sign or Symptom**	0.837	0.859
**Non-traditional risk factor**	0.869	0.880
**Average**	0.875	0.886

PhenoNorm generally achieves very high levels of performance, which are always improved by the WordNet-based pre-processing step, and in some cases by a significant margin. The higher performance on literature articles is likely to be due to the more formal nature of the writing, in which authors frequently use more standardised forms that tend to closely resemble the concept synonyms listed in the UMLS Metathesaurus.

An analysis of the output of PhenoNorm confirms that it is able to handle a variety of types of variation in entity mentions, e.g., orthographic differences (*light-headedness* vs. *lightheadedness*), morphological differences (*hyperkalemia* vs. *hyperkalemic*), alternation of Roman and Arabic numerals (*type II diabetes* vs. *type 2 diabetes*), differing numbers of words (*mild mitral regurgitation* vs. *mitral regurgitation*), semantic synonyms (*worsening renal function* vs. *decreased renal function*) and different word ordering or internal structure of mentions (*jugular venous pressure is elevated* vs. *elevated jugular venous pressure*). Entity mentions containing spelling mistakes can also be handled, e.g., *left ventricular hypertrophi* is correctly normalised to *left ventricular hypertrophy*.

#### Literature article analysis

In the majority of cases, PhenoNorm makes the correct decisions in mapping phenotype entity mentions in literature articles to UMLS concepts, even when there are differences in the number and ordering of words in the closest concept synonym. For example, the mention *increasing chest pain* is correctly mapped to the UMLS synonym *chest pain increasing in severity*, while the mention *stenosis in left anterior descending* is mapped to the synonym *left anterior descending coronary artery stenosis*.

A small number of cases cannot be handled by PhenoNorm. Since the generation of semantic variations only operates on a word by word basis, it is impossible for the method to normalise the mention *increased oxygen requirement* to the UMLS concept synonym *hypoxia*. However, it is also the case that none of the baseline methods could handle such a mapping. Even though UMLS lists *oxygen deficiency* as a synonym for this concept, PhenoNorm actually maps *increased oxygen requirement* to *increased insulin requirement*, based on the similarities between their lengths and the fact that two words are shared. Even though, in terms of semantics, the two shared words are not the most important ones, PhenoNorm is not able to consider this.

There are also cases where matching particular words can be more important than considering overall length. For example, the mention *chronic leg edema* is mapped to the concept synonym *chronic leg ulcer* instead of *leg edema*. Although, in semantic terms, it is most important to map a mention to a concept that concerns the same disorder, the importance of overall length for PhenoNorm means that in this case, it actually chooses a synonym that includes the same qualifier term as the mention, but which describes a different disorder. Such problems are partly due to the inconsistency amongst concepts listed in UMLS; for some disorders, there are specific concepts corresponding to qualified versions of the disorder, whilst in other cases, there is only a concept corresponding to the general disorder. Because of this, it may be advantageous to investigate a different method of assigning similarity scores, according to the *types* of words that are matched by the method, e.g., matching nouns may be more important than matching adjectives.

#### EHR analysis

In narrative content from EHRs, the performance of PhenoNorm in normalising entity mentions belonging to the *Cause* and *Non-traditional risk factor* semantic categories is almost as high as for literature articles, because such concepts are mentioned in quite standardised ways across both text types. For example, mentions of *Cause* concepts often correspond to disease names, e.g., *hypertension* and *mitral regurgitation*, which are rarely qualified with additional words or expressed using complex syntactic structures in either text type.

The results are somewhat lower for *risk factor* and *sign or symptom* in narrative EHR reports, which appears to be due to their frequent occurrence as long phrases, usually accompanied by qualifiers (e.g., *increased*, *reduced*, *elevated*, *moderate*, *severe*, etc.). Although the design of PhenoNorm aims to account for potential presence of such qualifiers, there is a wide range of possible qualifier terms, which are often not provided amongst the lists of synonyms of qualified concepts in UMLS. However, our WordNet-based pre-processing step, which targets such variation, can be very helpful in increasing the accuracy of the normalisation in such cases (by up to 4.6% for *sign or symptom* entity mentions in narrative EHR reports).

Another type of mapping error can occur when, even though the phenotype mention contains all of the words in a UMLS concept synonym, it should actually be mapped to a synonym of a different concept. An example is the mention *family history for coronary heart disease*, which is mapped to the UMLS concept synonym *family history*: *premature coronary heart disease* instead of to *family history of coronary artery disease*. This highlights the potential need to investigate more sophisticated methods of discovering semantic similarities between the terms being compared, e.g., to allow a link between *heart* and *artery* to be established. A possible solution would be to explore the use of more complex semantic relations that are encoded in WordNet, such as *meronomy* and *holonomy*, which deal with part-whole relationships between word referents.

### Baseline method evaluation

Table 3 compares the best overall results achieved by PhenoNorm when applied to PhenoCHF (i.e., using WordNet-driven semantic variant generation), with the results achieved by the baseline methods. For both text types, PhenoNorm achieves higher performance than any of the baseline methods, thus demonstrating its superior handling of the different types of variability that is inherent in phenotype entity mentions.

**Table 3:** Comparison of MetaMap, BeCAS, SoftTFIDF and PhenoNorm when applied to PhenoCHF.

Method	Accuracy	
	Narrative EHR reports	Articles
**MetaMap**	0.469	0.631
**BeCAS**	0.187	0.353
**SoftTFIDF**	0.764	0.837
**PhenoNorm**	0.847	0.886

In both MetaMap and BeCAS, the steps of NER and normalisation are essentially combined. Thus, if these tools fail to match a phenotype entity mention against a UMLS concept synonym, then no entity mention will be recognised and hence no normalisation will take place. Accordingly, we count as incorrect normalisations *both* a) cases in which the tool has recognised an entity mention whose text span is (partially) the same as an annotated mention in PhenoCHF, but where the recognised mention is mapped to the incorrect UMLS concept and b) cases where the tool does not recognise an entity mention that is annotated in PhenoCHF. In contrast, since, like PhenoNorm, SoftTFIDF takes as its starting point the annotated entities in PhenoCHF, it will always produce a mapping for each entity mention annotated in PhenoCHF.

It is also important to note that the performance of BeCAS cannot be directly compared to the other two methods, because it recognises disorders based on the SNOMED-CT [17] definition of the *disorder* semantic group which, in contrast to the definition used in the UMLS Metathesaurus, excludes the *finding* semantic type. However, a significant proportion of phenotype entity mentions annotated in PhenoCHF describe concepts that belong to the *finding* semantic type.

SoftTFIDF achieves the highest performance amongst the baseline methods, which is fairly close to the performance of PhenoNorm for literature articles. However, the lower results achieved for narrative EHR reports demonstrate that this method is not sufficiently robust to handle the wider variability in entity mentions encountered in this text type.

The considerably lower results achieved by MetaMap and BeCAS occur because they can essentially only recognise and normalise entity mentions corresponding to simple noun phrases that include the same words as those found UMLS concept synonyms (although possibly with a different ordering). Accordingly, they are usually unable to correctly recognise and normalise mentions of more complex concepts, and instead split them into a number of simpler mentions, each of which is mapped (incorrectly) to a separate concept. For example, in encountering the mention *stenosis in left anterior descending*, MetaMap detects three separate concepts, i.e., the more general medical condition *stenosis*, the spatial concept *left anterior* and the qualitative concept *descending*. Whilst BeCAS behaves in a similar way, it also separately recognises phrases that correspond to anatomical entities. For example, *Three Vessel Coronary Artery Disease* is recognised as two concepts, i.e., *Three Vessel* and *Coronary Artery Disease*.

Entity mentions containing words whose forms are different to those in concept synonyms within UMLS will also fail to be recognised by MetaMap and BeCAS. For example, the mention *bradycardiac* is not recognised by BeCAS as a variant of the concept synonym *bradycardia*. Problems also occur if the entity mention contains words that do not appear in the listed UMLS concept synonyms. For example, if qualifier terms occurring in entity mentions do not match the qualifier terms within any of the listed synonyms of a UMLS concept, then it is likely that the entity mention will be incorrectly mapped by MetaMap and BeCAS to a more general concept. So, the mention *high jugular venous pressure* (whose closest concept synonym in UMLS is *raised jugular venous pressure*) will be mapped to the more general *jugular venous pressure*.

Errors made by SoftTFIDF occur mainly because of its assignment of the highest similarity score to the shortest UMLS concept synonym with the highest number of exact (or similar) words shared with the entity mention. This means that it will often miss important qualifier concepts that determine the degree of the condition or sign/symptom (e.g., *high*, *low*, *moderate*, *severe*), if they are specified in the entity mention using different words to those used in any of the synonyms associated with the concept in the UMLS Metathesaurus. Accordingly, mapping may occur to a more general concept. For example, the phenotype mention *moderately reduced left ventricular systolic function* is mapped to the concept synonym *moderate left ventricular systolic dysfunction* instead of *moderately or severely depressed left ventricular systolic function*. Although non-exact matches between words can be handled, e.g., to pair *moderately* in the entity mention with *moderate* in the UMLS concept synonym, the qualifier terms *reduced* and *depressed* are not sufficiently similar to achieve a match, and semantic-level similarities are not considered by this method. In contrast, PhenoNorm carries out the correct mapping, since its initial selection of candidate UMLS concept synonyms considers synonyms that are longer as well as shorter than the entity mention. Then, by considering overall length and edit distance, *PhenoNorm* is able to correctly choose the UMLS synonym that includes the qualifier information.

In contrast to MetaMap and BeCAS, however, SoftTFIDF always considers the whole entity mention when mapping (even when the mention corresponds to a complex concept described using a long phrase). Since its “relaxed” token matching strategy is able to match words that have different forms to those included in UMLS concept synonyms, more accurate normalisation performance can be achieved than the terminology-driven approaches.

### Comparison of concepts used in literature articles and EHR narratives

**Fig 2:**
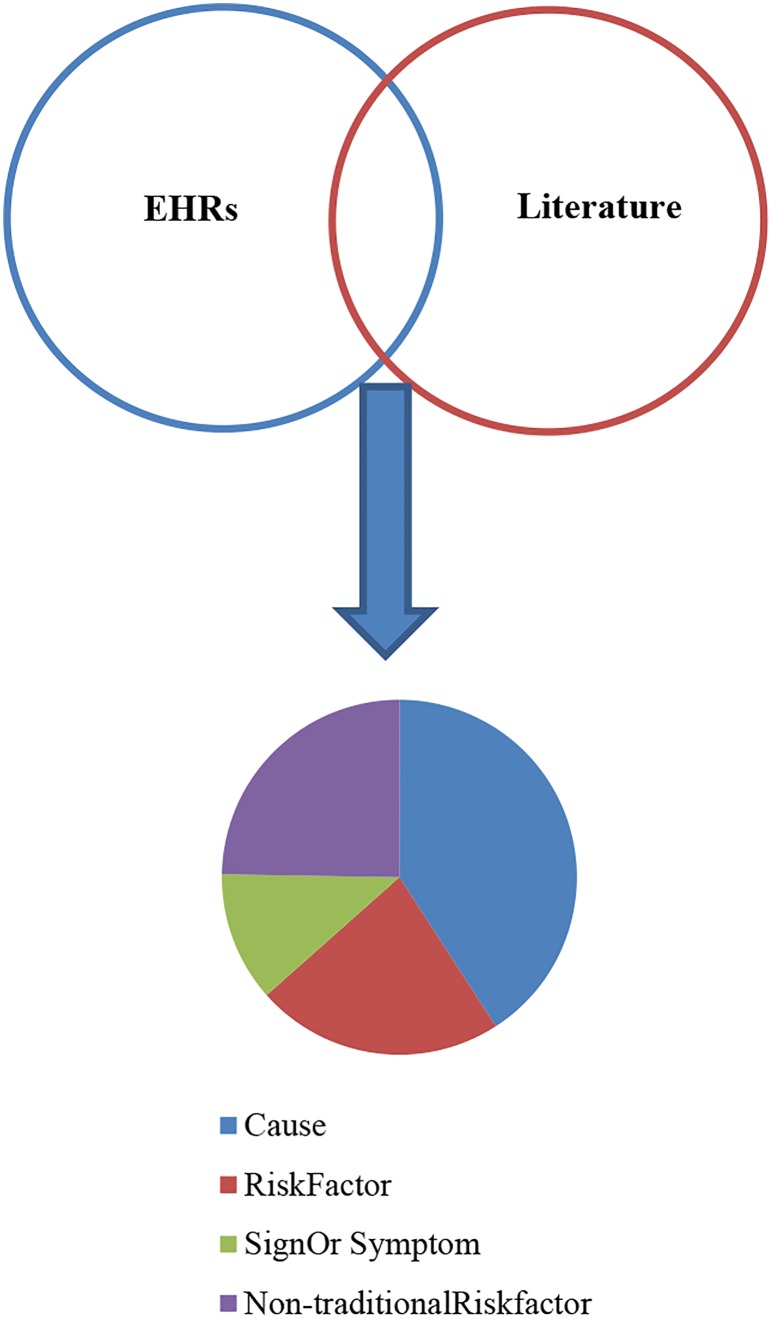
The overlap between phenotype concepts appearing in EHR narratives and literature articles.

The gold standard normalisations of entity mentions in PhenoCHF reveal that 835 concepts are mentioned in the corpus as a whole. Of these, 184 occur in both narrative EHR reports and literature articles, as shown in Fig 2.

The dominant phenotype concept type in narrative EHR reports is *Signs or symptoms*, whereas in the literature articles, there is far greater emphasis on discussing *Causes* [4]. However, the frequent mention of possible causes of observed signs and symptoms in narrative EHR reports, combined with the fact that that literature articles will often summarise these potential causes, helps to explain why *Cause* is the phenotype entity type with the greatest overlap between narrative EHR reports and literature articles.

The fact that the remaining 651 concepts are only mentioned *either* in EHR narrative reports or literature articles (but not in both) provides strong evidence of the complementary nature of the information contained within the two text types, and the need to combine this information to increase the opportunities of discovering new knowledge and generating novel hypotheses. However, our finding that there is a significant overlap between the mentions of concepts that occur in both parts of the corpus provides evidence that common types of information are reported in the two text types. The shared concepts could form the basis of establishing links between information in literature articles and EHR narrative reports, thus making it easier to combine information from the two sources in a sensible way. A further way of exploiting the links would be to provide personalised healthcare by finding connections between a patient’s EHR and new findings/ discoveries in the literature.

Some examples of the different ways in which concepts are mentioned in the two text types are shown in Table 4. The fact that this divergence of expression between the two text types is prevalent in mentions of shared concepts helps to emphasise the strong need to employ sophisticated normalisation methods such as PhenoNorm, if links are to be successfully established between the two information sources.

**Table 4:** Examples of different ways of mentioning the same phenotype concepts in narrative EHR reports and literature articles.

Type of variability	PhenoCHF corpus	
	EHR narrative mentions	Article mentions
**Synonymy**	Sodium overload Drop in blood pressure	Hypernatremia Hypotension
**Syntactic structure**	Left ventricle is dilated Mild mitral calcification	Left ventricular dilatation Calcification of mitral valve
**Word ordering**	Cardiac output decreased	Decreased cardiac output
**Morphological variation**	Hyperkalemic	Hyperkalemia
**Additional qualifier word**	Moderate left ventricular enlargement	Left ventricular enlargement

## Evaluation of PhenoNorm on Other Normalisation Tasks

To demonstrate that PhenoNorm is useful in a wider range of scenarios than only normalising mentions of phenotype concepts relating to CHF, we have evaluated its performance when applied to other normalisation tasks. Specifically, we have applied the method to four different corpora of biomedical documents that include gold-standard annotations of both entity mentions and links to concepts in terminological resources, i.e., the ShARe/CLEF corpus, the NCBI disease corpus [34], and the recently released corpora containing phenotype annotations relating to heart failure and pulmonary embolism [73]. These were chosen according to their different characteristics compared to PhenoCHF, i.e., either their documents belong to different text types and/or cover different subject areas to PhenoCHF, they are annotated with entity mentions belonging to semantic categories that are different to those annotated in PhenoCHF, and/or the entity mentions are mapped to concepts in terminological resources other than the UMLS Metathesaurus. To facilitate comparison between the performance of PhenoNorm and other normalisation approaches previously applied to the same corpora, we have used the metrics originally reported in evaluating these alternative approaches. Below, we provide brief descriptions of each data set and analyse the results obtained by applying the PhenoNorm method to each of them.

### ShARe/CLEF corpus

The ShARe/CLEF corpus is a collection of 300 narrative clinical records annotated for disorder mentions, which are linked to concepts in the UMLS Metathesaurus [11]. Although the types of entity mentions annotated in this corpus have much in common with those annotated in PhenoCHF, the subject areas covered by the narrative clinical records in the ShARE/CLEF corpus are wider, as is the semantic scope of the entity mentions annotated.

PhenoNorm achieved an accuracy of 0.83 on the ShARe/CLEF corpus, which is only slightly lower than its accuracy when applied to narrative clinical records in PhenoCHF. Many of the mapping errors made by PhenoNorm when applied to the ShARe/CLEF corpus can be explained by the specific characteristics of some of the annotations in this corpus. In particular, a large number of challenging abbreviations and acronyms are annotated, some of which are ambiguous, e.g., *3VD*, which PhenoNorm normalised to *three vessel disease*, but which in the gold standard is normalised to *triple vessel disease of the heart*. Furthermore, in assigning gold standard links between entity mentions and concepts, annotators of the ShARe/CLEF corpus took into account the textual context of entity mentions. Thus, annotated entity mentions in isolation may not exactly correspond to the UMLS concepts to which they are linked. This is problematic for our method, since it considers only the entity mention, rather than the surrounding context. As an example, the ShARe/CLEF corpus contains the entity mention *recurrent ventral hernia*, which exactly matches a UMLS concept synonym, and hence this is the mapping chosen by PhenoNorm. However, the mapping assigned in the gold standard is to the more specific *recurrent ventral incisional hernia*.

Normalisation results for several other systems have been reported in the context of the ShARe/CLEF Task 1 in the 2013 eHealth Evaluation Lab (for which the ShARE/CLEF corpus was originally developed), in which participant systems were expected to carry out *both* NER *and* normalisation of the recognised entity mentions. Since the assessment of normalisation performance in this shared task was not completely decoupled from NER performance, we cannot directly compare the normalisation performance of other systems to that of PhenoNorm. However, to provide a general estimation of normalisation performance, we provide in Table 5 the NER performance and the normalisation accuracy for the correctly recognised entities of three systems that used alternative normalisation strategies to PhenoNorm, i.e., terminology-driven (MetaMap) [88], rule-based [56] and machine learning (DNorm) [89]. The provision of the two performance measures for each system helps to estimate their overall normalisation performance.

**Table 5:** Comparison of PhenoNorm against other approaches applied to the ShARe/CLEF corpus.

Method	NER Performance (F-score)	Normalisation accuracy of recognised entity mentions
**PhenoNorm**	-	0.83
**Metamap**	0.42	0.94
**Rules**	0.68	0.87
**DNorm**	0.85	0.90

NER performance is reported in terms of F-Score, which is calculated as

F-Score = 2 x ( (Precision x Recall) / (Precision + Recall) )

Where

Precision = TP / (TP+FP)

Recall = TP / (TP+FN)

TP is the number of *true positives*, i.e., cases where an entity mention recognised by the system matches the gold standard annotation. FP is the number of *false positives*, i.e., cases in which the system recognised an entity mention incorrectly. FN is the number of *false negatives*, i.e., cases where the system should have recognised an entity mention, but it did not. *Precision* measures the extent to which the entity mentions recognised by the system were actually correct; recall measures the extent to which the system recognised all of the entity mentions that it was supposed to recognise. F-score is the harmonic mean of precision and recall, which provides an overall performance measure for the system.

The highest normalisation performance on correct, automatically recognised entity mentions, is achieved by MetaMap. However, this is to be expected, since recognised entity mentions are normalised as an integral part of the NER process. Thus, if an entity mention has been recognised correctly, then it is also likely to have been normalised correctly. However, the low NER performance confirms our previous findings that MetaMap struggles to recognise more complex entity mentions. Hence, the correct normalisations are likely to concern only simple entity mentions. The rule-based method also achieves high normalisation performance for correctly recognised entity mentions. However, the fact that NER performance only reaches 0.68 F-Score means that the ability of the method to normalise certain types of entity mentions in the corpus is not being assessed. In contrast, the high NER performance achieved by DNorm, accompanied by its ability to normalise these recognised entity mentions to a high degree of accuracy, suggests that it is a very flexible method for both NER and normalisation. The fact that DNorm is based on machine learning helps to explain this superior level of performance.

### NCBI disease corpus

The NCBI disease corpus [34] consists of 793 PubMed abstracts, annotated for 6,892 disease mentions. In contrast to the other corpora compared, normalisation does not involve mapping entity mentions to concepts in the UMLS Metathesaurus, but rather to concepts in a different terminological resource, i.e., the MEDIC vocabulary [15], which merges Online Mendelian Inheritance in Man (OMIM) [18] into the disease branch of the MeSH controlled vocabulary. The annotated entity mentions represent a total of 790 unique disease concepts.

We evaluated PhenoNorm in terms of its ability to normalise disease mentions in the test subset of the NCBI corpus (100 abstracts and 960 disease annotations) to the most similar disease concept in the MEDIC resource. The results are shown in Table 6, in which a comparison is made with the normalisation methods applied by Leaman et al. [62] to the same data set. We report results in terms of F-Score, to allow the performance of PhenoNorm to be compared with other normalisation methods that have also been applied to the NCBI disease corpus. We also report the accuracy of PhenoNorm when applied to this corpus, to facilitate direct comparison of the performance of PhenoNorm on other corpora.

**Table 6:** Micro-averaged performance comparison of PhenoNorm against other normsalisation approaches applied to the NCBI disease corpus.

Method	F-score	Accuracy
**PhenoNorm**	0.69	0.64
**Norm**	0.33	-
**MetaMap**	0.57	-
**Inference method**	0.59	-
**Cosine similarity**	0.67	-
**DNorm**	0.78	-

To calculate the performance of PhenoNorm in terms of F-Score, we determined TPs, FPs and FNs as follows:

1. Each case of a correct normalisation is counted as a TP.
2. Each case where an entity mention corresponds to a single concept, and where PhenoNorm mapped the mention to a single incorrect concept, is counted as an FN.
3. Each case where an entity mention corresponds to a single concept, but where PhenoNorm mapped this to two different (incorrect) concepts is counted as 1 FP and 1 FN. For example, PhenoNorm mapped the entity mention *breast and ovarian cancer* to two separate concepts, i.e., *breast cancer* and *ovarian cancer*, but the gold standard mapping is to a single concept, i.e., *Hereditary Breast and Ovarian Cancer Syndrome*.

An analysis of the results of applying PhenoNorm to the NCBI disease corpus show that its ability to handle different word orders and non-exact matches of tokens are once again advantageous in this context, e.g., in allowing the disease entity mention *familial neurohypophyseal diabetes insipidus* to be mapped to the correct concept synonym in MEDIC, i.e., *Diabetes Insipidus*, *Neurogenic*.

However, the results achieved by PhenoNorm on this corpus are somewhat lower than its performance when applied to the PhenoCHF and the ShARe/CLEF corpora. This can be explained partly by the fact that normalisation of entity mentions in the NCBI disease corpus is being carried out to a completely different terminological resource, i.e., the MEDIC vocabulary. Additionally, the strategy followed by annotators to create the gold standard, as well as certain features of the entity mentions, complicate the normalisation process. For example, disease mentions that could correspond to a complete family of diseases are mapped in the gold standard to the more general concept in MEDIC. This means that, e.g., the entity mention *complement deficiency* is mapped in the gold standard to the more general concept *Immunologic Deficiency Syndromes*. PhenoNorm cannot handle mapping that requires such additional reasoning. According to the configuration of PhenoNorm to map mentions to concept synonyms with similar length, the mention *complement deficiency* is mapped by PhenoNorm to the concept synonym *C9 Deficiency*.

An additional difficulty concerns coordinated noun phrases. In some cases, they should be split and mapped to separate concepts, which PhenoNorm can handle correctly. For example, the phrase *breast*, *brain*, *prostate and kidney cancer* is correctly mapped by PhenoNorm to 4 separate MEDIC concepts, i.e., *breast neoplasms*, *brain neoplasms*, *prostatic neoplasms* and *kidney neoplasms*. Indeed, PhenoNorm can sometimes produce mappings that are more correct than those produced by the best-performing DNorm method, which incorrectly mapped this coordinated phrase to a single concept, i.e., *prostate cancer/brain cancer susceptibility*. In other cases, however, concept synonyms in MEDIC actually correspond to coordinated noun phrases, e.g. *breast and ovarian cancer syndrome* is a single concept synonym. However, since PhenoNorm always splits up coordinated phrases, in this case into *breast cancer syndrome* and *ovarian cancer syndrome*, the correct mapping will not be achieved. An additional problematic example also concerns a coordinated noun phrase, i.e., *familial and sporadic cancers*, which PhenoNorm mapped to two separate concepts, i.e., *familial cancers* and *sporadic cancers*. However, in the gold standard, the rule concerning mapping of such phrases to a more general concept was applied, such that the mention was actually mapped to the concept *neoplasms*.

The results show that in terms of F-Score, PhenoNorm achieves superior performance to all methods compared, apart from DNorm (which was also applied to the ShARe/CLEF corpus, as detailed in the previous section). It should be noted, however, that in contrast to PhenoNorm, the other approaches reported are based on performing *both* NER and normalisation. As has been previously mentioned, MetaMap performs NER and normalisation as an integrated process; this is also the case for Norm. The other three methods perform normalisation on the output of the BANNER NER system [9]. This was the same system used to carry out NER for disorder mentions for DNorm on the ShARe/CLEF corpus reported in Table 5. Thus, its high performance in recognising such mentions has already been demonstrated.

Similarly to the PhenoCHF corpus, the performance of MetaMap on the NCBI disease corpus falls behind that of PhenoNorm, although by a smaller margin than in the results reported for PhenoCHF. This may be because, as we have observed in PhenoCHF, there tends to be less variability in mentions of disease names than some other entity types, and particularly so in more formal text. The Norm tool [90] deals with variation in entity mention by normalising case, plurals, inflections and word order. However, its low performance suggests that accounting only for a restricted set of mainly grammatical differences between the entity mention and the concept synonym is not sufficiently flexible. Both the inference and cosine similarity methods are string similarity metrics. The inference method [63] works by applying a combination of rules that use the O (ND) difference string similarity algorithm [91]. The cosine similarity method achieves results that are most comparable to those achieved by PhenoNorm, for similar reasons to the comparable performance achieved by SoftTFIDF. As was the case for the ShARe/CLEF corpus, the DNorm method outperforms the other methods by a considerable margin, probably because of its very different approach to the problem. Specifically, it is based on machine learning-based and uses pairwise learning to learn the level of similarity between the disease mentions and concept synonyms in MEDIC. Taking into account that the F-scores reported in Table 6 combine NER performance and normalisation, the performance of DNorm on this task can be considered to be roughly equivalent to its ability to normalise disorder mentions in the ShARe/CLEF corpus.

Despite the superior performance of DNorm, it is worth noting that, since PhenoNorm was the best performing method amongst those that are not machine-learning based, it represents an attractive option when no gold standard normalisations are available to train machine learning methods.

### Heart failure and pulmonary embolism corpora

As has been explained earlier, Wang et. al. [73] have recently developed corpora of formal biomedical text (including textbooks, evidence-based online resources, practise guidelines and journal articles) pertaining to three different diseases (i.e., heart failure, rheumatoid arthritis and pulmonary embolism), which are annotated with different types of phenotype-related entity mentions (i.e., causes, sign or symptoms, diagnostic tests and treatments). The corpora were annotated in a semi-automatic way (i.e., automatic pre-annotation was verified and/or edited by domain experts). The annotation included both the identification and semantic categorisation of entities, and mapping of these entity mentions to concepts in the UMLS Metathesaurus. The heart failure corpus includes 2588 annotated entity mentions, while the rheumatoid arthritis and pulmonary embolism corpora are annotated with 193 and 425 mentions, respectively. The mapping to UMLS concepts was mainly carried out by a single annotator, although a small sample was annotated by a second expert, allowing a normalisation inter-annotator agreement (IAA) of 0.84 F-score to be calculated.

We evaluated the ability of PhenoNorm to map entity mentions to the corresponding UMLS concepts in the two corpora with the largest number of annotated entity mentions (i.e., the heart failure and pulmonary embolism corpora). The results are shown in Table 7, using the same method as was detailed above for the NCBI disease corpus to calculate TPs, FPs and FNs for PhenoNorm, and thus to allow its performance to be reported in terms of F-score. Once again, we also report the performance in terms of accuracy, to facilitate comparison with the other applications of PhenoNorm.

**Table 7:** Results of applying PhenoNorm to the heart failure and pulmonary embolism corpora.

Method	Corpus	F	Accuracy
**PhenoNorm**	**Pulmonary embolism**	0.76	0.77
**PhenoNorm**	**Heart failure**	0.83	0.86
**IAA between annotators**	**237 random mentions**	0.84	-

To the best of our knowledge, our work constitutes the first attempt to use these corpora to evaluate normalisation approaches. As such, we cannot compare our results with any other methods applied to these corpora. However, for reference, we compare our results with the IAA results mentioned above.

For the heart failure corpus, the normalisation accuracy achieved by PhenoNorm is almost as high as for the literature part of the PhenoCHF corpus, and for both corpora, the results are higher than those achieved on the NCBI corpus. This is likely to be because the types of entity mentions annotated in the heart failure and pulmonary embolism corpora correspond closely to those annotated in PhenoCHF, and also since normalisation is being carried out using the same terminological resource for which PhenoNorm was originally designed, i.e., the UMLS Metathesaurus.

In most cases, the design features of PhenoNorm were advantageous when carrying out normalisation of entity mentions in the heart failure and pulmonary embolism corpora. For example, the pre-processing step of splitting coordinated noun phrases into two or more phrases was again very useful, and allowed mapping of phrases such as *stable or unstable angina*, to appropriate separate concepts, in this case *stable angina* and *unstable angina*. PhenoNorm’s consideration of concept synonyms that have different lengths and word orders to the entity mentions also allowed mapping of mentions such as *permanent pacemaker implantation* to *Implantation of permanent intravenous cardiac pacemaker*.

However, similarly to the other corpora compared, the guidelines for the gold standard normalisation of entity mentions in the heart failure and pulmonary embolism corpora instructed annotators to map each phenotypic mention to the concept conveying the mention’s specific meaning within the context of the original sentence. As an example, the entity mention *continuous blood pressure monitoring* is mapped in the gold standard to *continuous sphygmomanometers*. Since PhenoNorm is unable to consider semantic level variations in which several words in the mention are mapped to a single word in the corresponding concept synonym, the mapping predicted by PhenoNorm is to the more general *blood pressure monitoring*.

## Conclusion

We have presented a novel method, PhenoNorm, for mapping mentions of phenotype concepts appearing in heterogeneous textual sources, i.e., narratives in EHRs and literature articles, to appropriate concepts in the UMLS Metathesaurus. To our knowledge, PhenoNorm is the first method that is specifically targeted at the normalisation of mentions of phenotype concepts. As our analysis has demonstrated, such mentions can exhibit considerable diversity, with significant differences observable between narratives in EHRs and literature articles, in terms of structural and word-level variations in the ways in which specific phenotype concepts can be mentioned. These variations include different orderings of words, different forms of words and the use of semantically related words. To address this potential variability, PhenoNorm combines different string-based and semantic-level similarity methods. The accurate normalisation results produced by PhenoNorm, which we have shown to be superior to those achieved by other normalisation methods when applied to the PhenoCHF corpus, for both narratives in EHRs and literature articles, constitute an important first step towards the effective integration of complementary information dispersed within these different text types, in order to facilitate new knowledge discovery and generation of new hypotheses.

The expert-verified gold standard normalisations to UMLS concepts that have been added to all entity mentions in PhenoCHF, with the aid of PhenoNorm, add value to the corpus, and will allow it to be used in future for the training and evaluation of novel machine learning approaches to normalising phenotype entity mentions.

The application of PhenoNorm to the ShAre/CLEF corpus, the NCBI disease corpus, and the heart failure and pulmonary embolism corpora, has demonstrated that PhenoNorm can also achieve competitive performance when the complexity and the parameters of the task (e.g., text type, subject area, entity types and terminological resource) are changed. The encouraging results achieved, which are highly competitive with the results achieved by various other normalisation methods applied to the same corpora, help to illustrate the potentially wide utility of PhenoNorm as a means to normalise various types of entity mentions in biomedical literature and narrative clinical text.

According to the slightly lower results achieved for the NCBI disease corpus, there may be a need to tune certain parameters of PhenoNorm when the underlying terminological resource is changed; we will investigate this as future work. We also intend to consider the integration of more sophisticated semantic-level similarity measures to further improve the accuracy of the normalisation performance.
